# Male gender and kidney illness are associated with an increased risk of severe laboratory-confirmed coronavirus disease

**DOI:** 10.1186/s12879-020-05408-6

**Published:** 2020-09-16

**Authors:** Efrén Murillo-Zamora, Xóchitl Trujillo, Miguel Huerta, Mónica Ríos-Silva, Oliver Mendoza-Cano

**Affiliations:** 1 Departamento de Epidemiología, Unidad de Medicina Familiar, No. 19, Instituto Mexicano del Seguro Social, Av. Javier Mina 301, Col. Centro, C.P.28000 Colima, Colima México; 2 Centro Universitario de Investigaciones Biomédicas, Universidad de Colima, Av. 25 de julio 965, Col. Villas San Sebastián, C.P. 28045 Colima, México; 3 Universidad de Colima - Cátedras CONACyT, Centro Universitario de Investigaciones Biomédicas, Av. 25 de julio 965, Col. Villas San Sebastián, C.P. 28045 Colima, México; 4 Facultad de Ingeniería Civil, Universidad de Colima, km. 9 carretera Colima-Coquimatlán, Col. Jardines del Llano, C.P. 28400, Coquimatlán, Colima, México

**Keywords:** COVID-19, Severe acute respiratory syndrome coronavirus 2, Humans, Male, Kidney diseases

## Abstract

**Background:**

To identify factors predicting severe coronavirus disease 2019 (COVID-19) in adolescent and adult patients with laboratory-positive (quantitative reverse-transcription polymerase chain reaction) infection.

**Method:**

A retrospective cohort study took place, and data from 740 subjects, from all 32 states of Mexico, were analyzed. The association between the studied factors and severe (dyspnea requiring hospital admission) COVID-19 was evaluated through risk ratios (RRs) and 95% confidence intervals (CIs).

**Results:**

Severe illness was documented in 28% of participants. In multiple analysis, male gender (RR = 1.13, 95% CI 1.06–1.20), advanced age ([reference: 15–29 years old] 30–44, RR = 1.02, 95% CI 0.94–1.11; 45–59, RR = 1.26, 95% CI 1.15–1.38; 60 years or older, RR = 1.44, 95% CI 1.29–1.60), chronic kidney disease (RR = 1.31, 95% CI 1.04–1.64) and thoracic pain (RR = 1.16, 95% CI 1.10–1.24) were associated with an increased risk of severe disease.

**Conclusions:**

To the best of our knowledge, this is the first study evaluating predictors of COVID-19 severity in a large subset of the Latin-American population. Male gender and kidney illness were independently associated with the risk of severe COVID-19. These results may be useful for health care protocols for the early detection and management of patients that may benefit from opportune and specialized supportive medical treatment.

## Background

Since its first detection in December 2019 [1], the coronavirus disease 2019 (COVID-19) caused by SARS-COV-2 (severe acute respiratory syndrome coronavirus 2) has spread rapidly to most regions of the world, including Latin America. In Mexico and by the first half of August 2020, more than 500 thousand confirmed cases and 55 thousand deaths had been registered [2].

The COVID-19 pandemic represents a major challenge for healthcare systems worldwide and the precise pathogenic mechanism of severe manifestations has not been elucidated, but it appears that primarily host factors rather than viral genetic mutations play a major role [3]. Clinical factors that had been associated with a greater risk for severe COVID-19 are numerous and include increasing age, pre-existing comorbidities (i.e. type 2 diabetes mellitus and kidney dysfunction), and biomarkers of end-organ dysfunction, among others [4].

Most published studies evaluating markers of severe illness have been conducted among Asian and European populations and identifying factors to predict disease severity in cases is fundamental to improve the survival rate from COVID-19 [5]. We aimed to identify what factors are associated with the risk of severe laboratory-confirmed COVID-19 among adolescent and adult patients in Mexico.

## Method

### Study design and setting

A retrospective cohort study was conducted in April 2020. Potential eligible subjects (laboratory-confirmed cases of COVID-19, quantitative reverse-transcription polymerase chain reaction, RT-qPCR) were identified from nominal records found in a national normative online system for respiratory viruses surveillance (RVSS). Eligible cases were registered at any of more than 1800 medical units (three levels of care) that the Mexican Institute of Social Security (*IMSS*, the Spanish acronym) has all across Mexico. The *IMSS* provides health care services to more than a third of the total population of Mexico [6].

### Study population

Individuals aged 15 years or older, with symptoms onset from February 28 to March 20, 2020, and with conclusive test results (SARS-COV-2 infection confirmed) were eligible. Individuals with missing clinical or epidemiologic data of interest were excluded. In Mexico, phase 2 of the COVID-19 pandemic started on March 24 and, before that, laboratory testing was performed in all suspected ambulatory and no ambulatory cases [7].

### Data collection

Demographic characteristics (sex, age), tobacco use (current), personal history of chronic communicable disease (HIV infection, no/yes) and noncommunicable disease (no/yes: obesity [body mass index of 30 or higher], arterial hypertension, type 2 diabetes mellitus, asthma, chronic kidney disease, immunosuppression, chronic obstructive pulmonary disease, or cardiovascular illness) were collected from the surveillance system. Date of healthcare-seeking and, when applicable, dates of hospital admission and discharge were also extracted from the audited database.

Additional clinical and epidemiologic data, such as if the influenza vaccine was applied during the same season as the onset of the acute illness (no/yes), and if acute symptoms were reported (cough, fever, headache, myalgia, arthralgia, odynophagia, chills, rhinorrhea, thoracic pain, diarrhea, polypnea, no/yes) were also extracted from the database. The primary data sources were the medical records of the enrolled patients; these records were obtained from the employed surveillance resource system.

### Outcome

Laboratory-confirmed COVID-19 patients, and for epidemiological purposes, were classified as severe if they reported dyspnea [8] that resulted in hospital admission. Patients with severe manifestation were the main binary outcome (no/yes). Patients without dyspnea, despite being admitted to hospital, were considered as non-severe COVID-19 cases.

### Laboratory methods

According to normative standards [7], clinical specimens (nasopharyngeal or deep nasal swab) are analyzed (SuperScript™ III Platinum™ One-Step qRT-PCR Kits) at any of four specialized regional laboratories integrated with the *IMSS* network for epidemiologic surveillance. The laboratory methods employed in the *IMSS* network follow strict quality assurance standards in the diagnosis of viral respiratory pathogens [9].

### Statistical analysis

Summary statistics were calculated, and the significance level was set at 5%. Risk ratios (RRs) and 95% confidence intervals (CIs), estimated by using generalized linear regression models, were employed to evaluate the association between the exposures to the analyzed risk, and the risk of severe COVID-19. All analyses were conducted using Stata version 14.0 (StataCorp).

### Ethical considerations

The Local Research Ethics Committee approved this study at the Mexican Institute of Social Security (*IMSS*, the Spanish acronym). With the Research Ethics Approval Number/ID: R-2020-601-015.

## Results

**Fig. 1:**
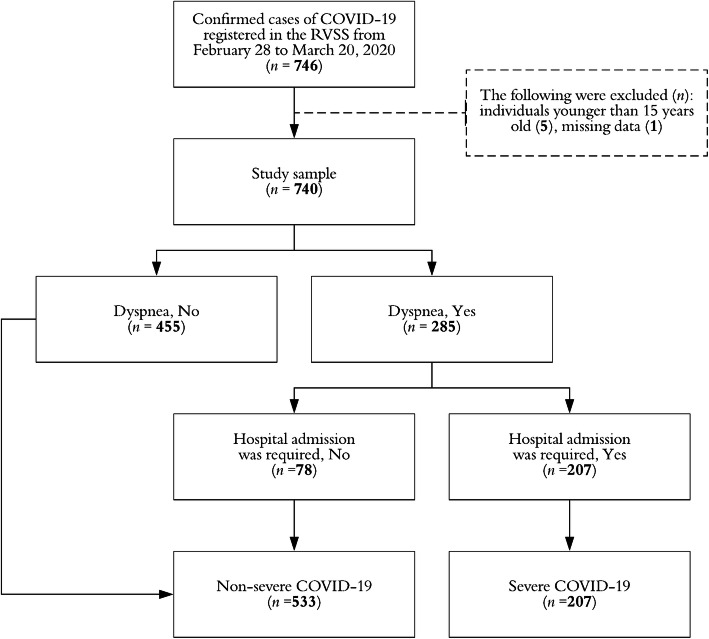
Study profile, Mexico 2020. Abbreviations: RVSS, Respiratory Virus Surveillance System; COVID-19, Coronavirus disease 2019

Data from 740 participants registered by 229 medical units located all across the country was analyzed. The study profile is shown in Fig. 1. Dyspnea was registered in 285 individuals, and 207 of them required hospital admission. Therefore, they were classified as severe COVID-19 cases (28.0% from enrolled subjects).

Table 1 shows the characteristics of the study sample for the variables selected. Most of the participants were male (57.3%), and the overall mean age (± standard deviation) was 43.7 ± 14.9 years old. No gender-related differences were observed in terms of age (*p* = 0.371). Two-thirds of enrolled patients were aged 30–59 years old. Antipyretic drugs were prescribed to most (55.9%) subjects before they sought healthcare and, in 9 out of 10 them, acetaminophen was consumed. The mean length of hospital stay in severe cases was 4.9 ± 4.1 days and ranged from 0 to 19 days.

**Table 1:** Characteristics of analyzed individuals with laboratory-positive non-severe and severe COVID-19, Mexico 2020

	Overall	Non-severe	Severe	***p***
	*n =* 740	*n* = 533	*n* = 207	
Sex				
Female	316 (42.7)	257 (48.2)	59 (28.5)	< 0.001
Male	424 (57.3)	276 (51.8)	148 (71.5)	
Age (years) ^a^	43.7 ± 14.9	40.0 ± 13.0	53.1 ± 15.4	< 0.001
Age group (years)				
15–29	141 (19.1)	126 (23.6)	15 (7.2)	< 0.001
30–44	281 (38.0)	240 (45.1)	41 (19.8)	
45–59	210 (28.3)	122 (22.9)	88 (42.6)	
≥ 60	108 (14.6)	45 (8.4)	63 (30.4)	
Received NAIs (any)				
No	734 (99.2)	529 (99.2)	205 (99.0)	0.769
Yes	6 (0.8)	4 (0.8)	2 (1.0)	
Received antyperitc drugs (any)				
No	326 (44.1)	227 (42.6)	99 (47.8)	0.198
Yes	414 (55.9)	306 (57.4)	108 (52.2)	
Antipyretic drug received ^b^				
Acetaminophen	372 (89.9)	291 (95.1)	81 (75.0)	< 0.001
Metamizole	11 (2.7)	5 (1.6)	6 (5.6)	
Ibuprofen	10 (2.3)	4 (1.3)	6 (5.6)	
Other	21 (5.1)	6 (2.0)	15 (13.8)	
Received influenza vaccine ^c^				
No	622 (84.1)	440 (82.6)	182 (87.9)	0.073
Yes	118 (15.9)	93 (17.4)	25 (12.1)	
Elapsed days from symptoms onset to health-care seeking				
< 1	347 (46.9)	253 (47.5)	94 (45.4)	< 0.001
1–3	182 (24.6)	148 (27.7)	34 (16.4)	
4 or above	211 (28.5=	132 (24.8)	79 (38.2)	
Hospital admission				
No	470 (63.5)	470 (88.2)	0 (0)	< 0.001
Yes	270 (36.5)	63 (11.8)	207 (100)	
***Acute symptoms profile***				
Cough (yes)	657 (88.8)	466 (87.4)	191 (92.3)	0.061
Fever (yes)	641 (86.6)	451 (84.6)	190 (91.8)	0.010
Headache (yes)	632 (85.4)	464 (87.1)	168 (81.2)	0.041
Myalgia/Arthralgia (yes)	572 (77.3)	423 (79.4)	149 (72.0)	0.031
Odynophagia (yes)	407 (55.0)	310 (58.2)	97 (46.9)	0.006
Chills (yes)	403 (54.5)	287 (53.8)	116 (56.0)	0.591
Rhinorrhea (yes)	353 (47.7)	276 (51.8)	77 (37.2)	< 0.001
Thoracic pain (yes)	282 (38.1)	171 (32.1)	111 (53.6)	< 0.001
Diarrhea (yes)	137 (18.5)	104 (19.5)	33 (15.9)	0.262
Polypnea (yes)	27 (3.6)	5 (0.9)	22 (10.6)	< 0.001
***Personal history of***				
Tobacco use (current, yes)	80 (10.8)	47 (8.9)	32 (15.6)	0.009
Obesity (BMI 30 or higher) (yes)	162 (21.9)	100 (18.8)	61 (29.7)	0.001
Arterial hypertension (yes)	148 (20.0)	74 (13.9)	74 (35.6)	< 0.001
Cardiovascular disease (yes)	20 (2.7)	8 (1.5)	12 (5.9)	0.001
Type 2 diabetes mellitus (yes)	108 (14.6)	51 (9.5)	58 (27.8)	< 0.001
Asthma (yes)	33 (4.4)	22 (4.2)	10 (4.9)	0.676
COPD (yes)	27 (3.7)	9 (1.7)	18 (8.8)	< 0.001
Chronic kidney disease (yes)	13 (1.8)	3 (0.6)	10 (4.9)	< 0.001
Immunosuppression (yes)	19 (2.5)	8 (1.5)	10 (4.9)	< 0.001
HIV infection (yes)	5 (0.7)	3 (0.6)	2 (1.0)	0.549

*Abbreviations: COVID-19* Coronavirus disease 2019, *NAIs* Neuraminidase inhibitors, *COPD* Chronic obstructive pulmonary disease, ***HIV*** Human immunodeficiency virus

Notes: 1) SuperScript™ III Platinum™ One-Step qRT-PCR Kits were used to confirm the COVID-19; 2) The absolute and relative (%) frequencies are presented, unless otherwise specified. 2) *p*-value from chi-square o t-tests as corresponding

^a^ The arithmetic mean ± standard deviation is presented

^b^ Among 414 subjects (non-severe disease, *n* = 306; severe disease, *n* = 108) in whom the use of antipyretic drugs was documented before healthcare seeking

^c^ During the same influenza season than acute COVID-19 onset

Severe COVID-19 cases were older (53.1 ± 15.4 vs. 40.0 ± 13.0, *p* <  0.001) when compared with non-severe patients (Table 1), and particularly in the eldest age group (≥ 60 years old; 30.4% vs. 8.4%). Severe cases were also more likely to seek healthcare after four or more days after symptoms onset (38.2% vs. 24.8%, *p* <  0.001).

Regarding the acute symptoms profile, severe cases had a higher frequency of fever (91.8% vs. 84.6%, *p* = 0.010), thoracic pain (53.6% vs. 32.1%, *p* <  0.001) and polypnea (10.6% vs. 0.9%, *p* <  0.001). Significant and higher prevalences of pain-related symptoms (headache, myalgia, arthralgia, odynophagia) were documented in non-severe cases (Table 1).

In the multiple regression analysis (Table 2), male gender (RR = 1.13, 95% CI 1.06–1.20) and older patients ([reference: 15–29 years old] 30–44, RR = 1.02, 95% CI 0.94–1.11; 45–59, RR = 1.26, 95% CI 1.15–1.38; 60 years or older, RR = 1.44, 95% CI 1.29–1.60), subjects to thoracic pain (RR = 1.16, 95% CI 1.10–1.24) or chronic kidney disease (RR = 1.31, 95% CI 1.04–1.64) were also more likely to present severe COVID-19. On the other hand, in multiple analysis the history of obesity, tobacco use, cardiovascular or metabolic diseases, and pulmonary illness (namely asthma or chronic obstructive pulmonary disease) were not associated with the evaluated outcome.

**Table 2:** Factors associated with the risk of severe laboratory-confirmed COVID-19, Mexico 2020

	Bivariate analysis				Multiple analysis			
	RR	95% CI		***p***	RR	95% CI		***p***
Sex								
Female	1.00				1.00			
Male	1.18	1.10	1.25	< 0.001	1.13	1.06	1.20	< 0.001
Age (years)								
15–29	1.00				1.00			
30–44	1.04	0.96	1.13	0.354	1.02	0.94	1.11	0.585
45–59	1.37	1.25	1.49	< 0.001	1.26	1.15	1.38	< 0.001
≥ 60	1.61	1.45	1.79	< 0.001	1.44	1.29	1.60	< 0.001
Received antyperitc drugs (any)								
No	1.00				1.00			
Yes	0.96	0.90	1.02	0.198	0.96	0.90	1.01	0.136
Elapsed days from symptoms onset to health-care seeking								
< 1	1.00				1.00			
1–3	0.91	0.85	0.99	0.039	0.96	0.89	1.02	0.213
4 or above	1.11	1.02	1.20	< 0.001	1.06	0.99	1.13	0.102
***Acute symptoms profile***								
Fever								
No	1.00				1.00			
Yes	1.13	1.03	1.25	0.010	1.07	0.98	1.17	0.109
Odynophagia								
No	1.00				1.00			
Yes	0.91	0.85	0.97	0.005	0.95	0.89	1.01	0.060
Myalgia/arthralgia								
No	1.00				1.00			
Yes	0.92	0.85	0.99	0.031	0.95	0.88	1.02	0.142
Thoracic pain								
No	1.00				1.00			
Yes	1.20	1.13	1.28	< 0.001	1.16	1.10	1.24	< 0.001
***Personal history of***								
Tobacco use (ever)								
No	1.00				1.00			
Yes	1.15	1.04	1.28	0.009	1.06	0.97	1.17	0.210
Obesity (BMI 30 or higher)								
No	1.00				1.00			
Yes	1.14	1.05	1.23	0.001	1.05	0.98	1.13	0.185
Chronic obstructive pulmonary disease								
No	1.00				1.00			
Yes	1.49	1.26	1.77	< 0.001	1.12	0.95	1.31	0.183
	**Bivariate analysis**				**Multiple analysis**			
	**RR**	**95% CI**		***p***	**RR**	**95% CI**		***p***
Arterial hypertension								
No	1.00				1.00			
Yes	1.32	1.22	1.42	< 0.001	1.05	0.97	1.14	0.207
Cardiovascular disease								
No	1.00				1.00			
Yes	1.39	1.14	1.69	0.001	1.09	0.91	1.31	0.372
Type 2 diabetes mellitus								
No	1.00				1.00			
Yes	1.34	1.23	1.47	< 0.001	1.06	0.96	1.16	0.239
Chronic kidney disease								
No	1.00				1.00			
Yes	1.65	1.29	2.10	< 0.001	1.31	1.04	1.64	0.020
Immunosuppression								
No	1.00				1.00			
Yes	1.33	1.08	1.64	0.008	1.14	0.94	1.37	0.180

*Abbreviations: COVID-19* Coronavirus disease 2019, ***RR*** Risk Ratios estimated through linear regression models, *BMI* Body mass index

Notes: 1) SuperScript™ III Platinum™ One-Step qRT-PCR Kits were used to confirm the CoViD-19; 2) The definition of severe COVID-19 included laboratory conclusive evidence of infection and dyspnea and hospital admission; 3) Risk ratios from multiple analysis were adjusted by the variables listed in the table

## Discussion

Our findings suggest that more than 1 of 4 analyzed patients developed severe COVID-19 and its predictors were identified. To the best of our knowledge, this is the first study evaluating factors associated with the illness severity in a large subset of Latin-American subjects.

Severe disease, according to our results, was presented by 28% (207/740) of enrolled laboratory-confirmed cases. This proportion is similar to government estimates (29%) at the national level (all healthcare institutions, including private facilities) by the end of the first week of April and when 3181 RT-qPCR-confirmed cases had been registered [10].

After adjusting by factors such as age, obesity and personal history of chronic noncommunicable diseases, males from our study sample were more likely (RR = 1.13, 95% CI 1.06–1.20) to develop dyspnea and to require hospital admission. Similar findings were previously described among Chinese adults, where male patients were more likely to develop severe COVID-19 [11].

Further research has to determine the pathogenic underlying mechanism between gender and the severity of SARS-COV-2 infection. Published data suggests that gender differences in susceptibility to SARS-COV (Severe Acute Respiratory Syndrome Coronavirus) in mice were secondary to estrogen receptor signaling, which seemed to be critical for protection in females [12]. However, the role of sex hormones in the regulation of innate immune cells in the lung, as a response to viral respiratory pathogens, is poorly understood [13].

In our study, a positive gradient between age and the risk of severe illness was observed (per additional year of age, RR = 1.010, 95% CI 1.007, 95% CI 1.012) in multiple regression analysis. Aging has been consistently associated with disease severity [14–16] and its determining factors have not been described but may be related to viral load. A recently published analysis showed a positive and strong correlation (Spearman’s *p* = 0.48, 95% CI 0.074–0.75) between older age and RT-qPCR quantified viral load [17]. Teenagers from our study sample (aged 15–19 years old, *n* = 5) seemed to have mild symptoms of SARS-COV-2 infection and all of them presented a non-severe form of disease.

Patients with previous medical diagnosis of chronic kidney disease had a 31% increase in the risk of severe COVID-19 (RR = 1.31, 95% CI 1.0–1.64). Interestingly, in our multiple regression study, neither type 2 diabetes mellitus (RR = 1.06, 95% CI 0.96–1.16) nor arterial hypertension (RR = 1.05, 95% CI 0.97–1.14), which are leading causes of renal impairment worldwide, were associated with the risk of severe COVID-19.

The association of previously diagnosed chronic kidney disease with the severity of COVID-19 was recently described in a subset of Chinese patients [5] When compared with national estimates (type 2 diabetes mellitus, 10.3%; arterial hypertension, 18.4%) [18], higher prevalence of chronic noncommunicable diseases were observed in the study sample, particularly in participants with severe manifestations (type 2 diabetes mellitus, 27.8%; arterial hypertension, 35.6%).

Mexico lacks of a national registry of chronic kidney disease patients and its precise prevalence remains unknown [19]. A previously published study where 3564 randomly selected adults residing in urban areas of Mexico were analyzed, estimated that the population prevalence of chronic renal disease (creatinine clearance, Cockcroft-Gault formula) was 8% (< 60 ml/minute) and 0.1% (< 15 ml/minute) respectively [20].

Thoracic pain was a common acute symptom and it was registered in medical records from 38.1% of enrolled subjects. It is a subjective symptom that seemed to be related to dyspnea, since it was presented by 55.1 and 27.5% of dyspneic and non-dyspneic patients respectively (*p* <  0.001).

The pathogenic mechanism of SARS-COV-2 in severe outcomes, including viral pneumonia, remains unclear; however, immunological changes seem to be crucial in the development of severe illness [21]. Increased levels of proinflammatory cytokines, particularly interleukin 6, have been described among patients with severe COVID-19 [22].

Current tobacco use, obesity, and personal history of chronic obstructive pulmonary disease were only associated with the risk of severe disease in bivariate analysis. The overall prevalence of tobacco use (current) in the study sample was similar to the national mean (10.8 and 11.4%, respectively) [18] and it was 37% higher among severe COVID-19 cases (15.6%). In China, there was a higher frequency for severe patients to be current smokers (16.9% vs. 11.8%), however, no association analysis was performed [23].

Obesity is assessed by the analyzed surveillance system as a dichotomous variable (body mass index (BMI) equal or higher than 30, no/yes) and the current consensus is that morbidly obese individuals (BMI ≥ 40) are at increased risk of severe COVID-19 [24]. Since the patients’ height and usual weight are not collected by the surveillance system, we were unable to compute de BMI and therefore to estimate the prevalence of severe obesity in the study sample.

Recently published data suggest the rapid decay of anti–SARS-CoV-2 immunoglobulin G (IgG) in early infection [25], and this decay seems to be faster among asymptomatic patients and those with mild COVID-19 [26]. These findings may be highly relevant to immunity strategies and other pandemic control strategies.

The potential limitations of our study must be cited. First, only users from a healthcare institution were enrolled (*IMSS*) and their characteristics may not be entirely representative of the source population. However, the profile of its users remains heterogeneous and SARS-COV-2 testing is provided for free to suspected COVID-19 cases.

Second, the system that served as a source of data focuses on epidemiological surveillance and clinical data (i.e. blood oxygen saturation, the ratio of the partial pressure of arterial oxygen to the fraction of inspired oxygen (PaO_2_:FiO_2_), or lung radiographic reports) are not systematically collected. Other relevant information, such as the personal history of malignant tumors, is neither obtained by this system. Most of the collected data is dichotomous (no/yes) in order to simplify its operation and we were unable to obtain other clinical and epidemiological data of interest. Besides, we analyzed a relatively small sample size and this may have an impact on the significance of multiple regression coefficients. Only cases occurring prior to March 24 (phase 2) were enrolled and this allowed us to reduce the risk of bias in analyzing only severe cases that were more likely to seek healthcare.

And third, the COVID-19 severity from an undetermined fraction of participants from our study may be misclassified given that we only used dyspnea requiring hospital admission to identify it. However, this stand-alone marker seemed to have an acceptable predictive value since invasive ventilator support was required in 0.5 and 7.5% of non-severe and severe cases (*p* <  0.001), respectively. A fatal outcome was documented in almost half (49.8%) of analyzed subjects with severe illness (vs. 10.2%; *p* <  0.001).

## Conclusions

The COVID-19 pandemic is a major public health issue across the world. An effective response of healthcare systems is needed, which must include early identification of patients who are at increased risk of severe symptoms and poorer disease outcomes. In our analysis, male gender and kidney disease were independently associated with an increased risk of severe respiratory illness. To the best of our knowledge, this is the first study evaluating predictors of COVID-19 severity in a large subset of the Latin-American population. These results may also be useful in medical decision making related to the start of SARS-COV-2 antiviral therapy, when available.

## Data Availability

All data generated or analyzed during this study are included in this published article and its supplementary information files.

## Abbreviations

COVID-19: coronavirus disease 2019
SARS-COV-2: severe acute respiratory syndrome coronavirus 2
RR: Risk ratio
CI: Confidence interval
RVSS: Respiratory Viruses Surveillance System
IMSS: Mexican Institute of Social Security (Spanish acronym)
RT-qPCR: quantitative reverse-transcription polymerase chain reaction
BMI: Body mass index
IgG: immunoglobulin G
PaO_2_: pressure of arterial oxygen
FiO_2_: fraction of inspired oxygen
